# Differential Effect of Retroactive Interference on Object and Spatial Memory in the Course of Healthy Aging and Neurodegeneration

**DOI:** 10.3389/fnagi.2018.00333

**Published:** 2018-10-23

**Authors:** Hannah Muecke, Nils Richter, Boris von Reutern, Juraj Kukolja, Gereon R. Fink, Oezguer A. Onur

**Affiliations:** ^1^Department of Neurology, University Hospital, Cologne University, Cologne, Germany; ^2^Department of Cognitive Neuroscience, Institute of Neuroscience and Medicine (INM-3), Research Centre Jülich, Jülich, Germany; ^3^Department of Neurology, Helios University Hospital Wuppertal, Wuppertal, Germany

**Keywords:** amnesia, consolidation, recognition, recall, hippocampus

## Abstract

**Objective**: In subjects with mild cognitive impairment (MCI), interference during memory consolidation may further degrade subsequent recall of newly learned information. We investigated whether spatial and object memory are differentially susceptible to interference.

**Method**: Thirty-nine healthy young subjects, 39 healthy older subjects, and 12 subjects suffering from MCI encoded objects and their spatial position on a 4-by-5 grid. Encoding was followed by either: (i) a pause; (ii) an interference task immediately following encoding; or (iii) an interference task following encoding after a 6-min delay. Type of interference (no, early, delayed) was applied in different sessions and order was counterbalanced. Twelve minutes after encoding, subjects saw objects previously presented or new ones. Subjects indicated whether they recognized the object, and if so, the objects’ position during encoding.

**Results**: Interference during consolidation provoked a negative effect on spatial memory in young more than older controls. In MCI, object but not spatial memory was affected by interference. Furthermore, a shift from fine- to coarse-grained spatial representation was observed in MCI. No differential effect of early vs. late interference (EI vs. LI) in either of the groups was detected.

**Conclusions**: Data show that consolidation in healthy aging and MCI differs from consolidation in young controls. Data suggest differential processes underlying object and spatial memory and that these are differentially affected by aging and MCI.

## Introduction

It is generally accepted that the hippocampus plays a key role in spatial memory (Holdstock et al., 2000). However, the extent of its involvement and the interplay with extra-hippocampal areas seem to depend upon the specific context. Whereas fine-grained representations of space rely on the (posterior) hippocampus with its place cells covering small fields (Brun et al., 2008), coarse-grained representations seem to depend less upon the hippocampus. Rather, these seem to rely on the parahippocampal and lingual gyri as well as the posterior cingulate, retrosplenial and lateral temporal cortices (Rosenbaum et al., 2004, 2007; Hirshhorn et al., 2012).

Converging evidence for this notion is provided by studies investigating patients with hippocampal lesions, which reveal disturbed fine-grained spatial memory while more coarse details may still be retrievable (Rosenbaum et al., 2000).

Consolidation is a challenging process to study as it happens unconsciously and experimental paradigms are scarce. One approach is to disturb consolidation with an interference task presented between encoding and retrieval, and, hence, to investigate if consolidation of object and spatial memories, fine-grained or coarse-grained, are differentially susceptible to interference. Furthermore, the timing of interference during consolidation may be relevant in terms of a stronger interference effect the earlier the interference occurs (Skaggs, 1933; Dewar et al., 2009).

As investigating participants with deficient memory due to a lesion or a neurodegenerative process is a promising approach to further our understanding of the process underlying memory consolidation, investigation of object memory and fine-grained spatial representations in subjects suffering from an amnestic mild cognitive impairment (aMCI; Petersen, 2004) seems warranted. Most patients with aMCI show a decay in delayed recall of newly learned information (Baddeley and Warrington, 1970), even if the delay is reduced to 1 min (Cowan et al., 2004).

Studying subjects with aMCI and healthy young and older controls in the same study imposes problems since a paradigm may be appropriate for healthy controls but too difficult for subjects with aMCI or it may be appropriate for the aMCI, while being too easy for the controls. In order to make interference effects comparable between groups, we adjusted difficulty levels individually.

We hypothesized that with normative aging and neurodegeneration the strength of interference on consolidation changes and that interference effects object and spatial memory differentially. For compensation, we expected a shift from fine to coarse-grained spatial representations (Rosenbaum et al., 2000). Concerning the timing of interference, we expected a greater impact of early (EI) rather than late interference (LI; Skaggs, 1933; Dewar et al., 2009).

## Materials and Methods

### Participants

In total, 98 adults participated in the study. Three participants abandoned the experiment before completion, four further subjects were excluded due to signs of depression and pathological scores in the respective tests, and one subject was excluded due to failure to understand the task correctly. Thus, 90 participants were included in the further analyses. In detail, we tested 12 subjects with aMCI (five females; mean age 69.2 years, SD 7.98, age range 56–81), 39 older controls (19 females; mean age 68.6 years, SD 6.25, age range 55–89), and 39 young controls (19 females; mean age 26.2 years; SD 2.26, age range 21–32) with matching sociodemographic and neuropsychological evaluation (except for aMCI). All participants had a normal neurological examination and were healthy according to their medical history. All participants gave informed written consent before participating, according to the Declaration of Helsinki. Ethics approval was obtained from the local ethics committee (Medical Faculty of the University Cologne, Germany).

### Neuropsychological Testing

In order to obtain a neuropsychological profile from each participant, every participant underwent neuropsychological testing, comprising the Verbal Learning and Memory Test (VLMT, Lux et al., 1999), which is, by its delayed recall of new learned information a valuable indicator of ongoing cognitive impairment (Bondi et al., 2008). We further used the Complex-Figure-Test (CFT by Rey-Osterrieth) for visuoconstruction (Pena-Casanova et al., 2009), the Edinburgh handedness inventory (McMeekan and Lishman, 1975), and the Becks depression inventory (BDI V) to detect signs of depression (Beck and Steer, 1984). In case the BDI V suggested depression, we performed the SKID (structured interview, Münster, 1999), which resulted either in a normal score with subsequent inclusion of the participant, or a pathological score with subsequent exclusion of the participant. Moreover, we performed the Brief test of attention (BTA, Schretlen et al., 1996) and the Trail-making Test (TMT) to assess attention, executive functions and task switching (Reitan, 1955), the Memory Assessment Clinics Questionnaire (MAC-Q) for self-evaluation of overall memory performance (Crook et al., 1992), the Leistungsprüfsystem, test 4 (LPS-4) for the intelligence level (Horn and Cattell, 1966), the Bayer Activities of daily Living Scale (Bayer-ADL, Hindmarch et al., 1998) to rule out impairments of activities in daily living, and the DemTect (Kalbe et al., 2004).

### Experimental Procedure

The experiment comprised four visits. At the first visit (baseline), neuropsychological testing was performed. In addition, test runs of the paradigm were performed with the aim to familiarize participants with the experimental set-up and the task and to assess the individual memory performance at baseline. Taking into account the relevant differences in memory capacity between healthy young, healthy older, and subjects with aMCI, we aimed at a performance level for the main experiment of 50%–75% correct responses in fine-grained spatial memory, in order to avoid ceiling or floor effects, and to ensure comparable task performance across subjects and groups. To this end, different levels of encoding task difficulty were used (level 1—the most difficult level, level 6—the easiest level). Encoding difficulty was modulated by changing the number of items to be memorized (3 to 18 objects) and by the number of repetitions with which individual objects were presented (two to four repetitions). In the pre-testing session, we started with level 4 for all participants. Depending on the test result and based on a predefined algorithm, participants performed a second run with a different level and a new set of stimuli. Based on the results of both runs a level of difficulty was chosen, which we presumed to result in an accuracy level of 50%–75% in fine-grained spatial memory. The main experiment encompassed three consecutive visits (each 1 week apart). During each visit, participants performed the same task at the same difficulty level but with different sets of stimuli.

**Figure 1:**
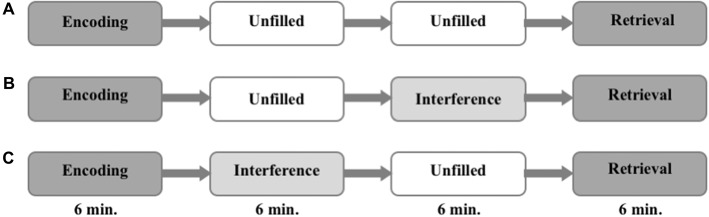
Experimental procedure. The order of interference mode **(A–C)** are randomized and counterbalanced within the groups.

The task was divided into three parts. It started with an encoding run and ended with a retrieval run. The phase in between differed from session to session. During this period either: (i) nothing happened and the subjects were asked to rest (no interference, NI); or (ii) an interfering task was presented immediately after the encoding (EI); or (iii) the interference task took place right before the retrieval (LI). The sequence of the different conditions was counterbalanced between the different groups (i.e., one third of participants started with NI, one third with EI, and one third with LI) to control for order effects. The encoding and the retrieval runs lasted 6 min each, and the consolidation period lasted 12 min. The interference task (EI and LI) during consolidation lasted 6 min (see Figure 1).

### Paradigm

#### Memory Task

**Figure 2:**
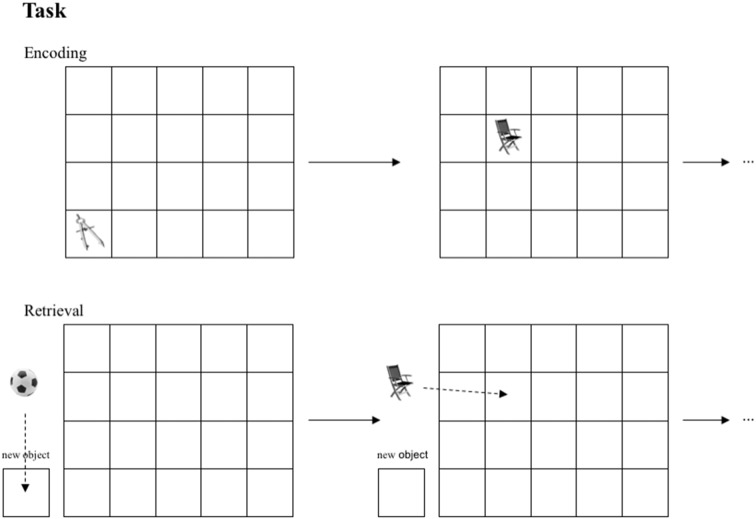
Task performed by all groups. In the encoding session objects are presented in sequential order in one of 20 fields. In the retrieval session subjects are asked to position learned objects in the corresponding fields.If an object was not presented in the encoding session, subjects were instructed to place this object into the box “new object” in the retrieval session.

The task used in this study is a modification of well-established spatial contextual memory tasks (Smith and Milner, 1981; Nunn et al., 1999; Cansino et al., 2002; Kukolja et al., 2009). Participants were instructed to memorize objects and their location in a grid of 20 fields (four-by-five), which was adapted to a computer screen. The stimuli were small photographs of either natural (e.g., a tree) or artificial/man-made (e.g., a shoe) objects. Every object was presented for 4.5 s. To ensure that subjects paid attention, participants had to distinguish between natural and artificial objects by pushing one of two marked keys on the keyboard (index finger of the right hand for natural and index finger of the left hand for artificial objects). The number of the to-be-learned objects depended on the level of the encoding task difficulty, which was established during the first visit (18 objects in level 1; 15 in level 2; 12 in level 3; 9 in level 4; 6 in level 5; and 3 in level 6). The fewer objects presented, the more often they were repeated in the same location (two repetitions in levels 1 and 2; three repetitions in levels 3 and 4; four repetitions in levels 5 and 6). The number of stimuli presented was almost equal across all groups and difficulty levels. Every location in the 4 × 5 grid was used once only to avoid interference at encoding. After a break of 12 min, either objects shown during the encoding session or new (i.e., not yet presented) objects in equal numbers were presented left to the grid. Participants had to indicate whether the object had been presented during encoding or not (“object memory”) using a computer mouse to move the stimuli on the screen without pressing any button. New objects had to be allocated to a highlighted zone outside the grid (“new object”). Objects were considered as successfully retrieved in terms of object memory whenever an object was positioned in the grid irrespective of the exact position. If the memorized object from the encoding session was placed into the square of the grid where the object had been presented during encoding, it was considered as correct response in terms of spatial memory (see Figure 2).

If the object was positioned exactly in the field in which it had been presented during encoding, the response was classified as correct fine-grained spatial context retrieval. If the object was placed one field to the left, right, up and down of the correct position, the response was accepted as correct coarse-grained spatial context retrieval. So, both object and spatial memory was assessed by one task through one response. Our task differed from the above-mentioned paradigms in following aspects: (1) our objects were presented in a sequential order; no more than one object was presented at the same time. (2) the stimuli were pictures of objects placed within in rectangle grid with 20 fields presented on a computer screen and were not real objects freely distributed on a piece of sheet.

The participant had 8.4 s to indicate each object’s position (stimulus was displayed for the whole period regardless of correct or incorrect positioning) followed by a pause of 4.2 s until the next object appeared. Since healthy older participants and subjects with aMCI are less used to handle a computer mouse to move objects on a computer screen, the above-mentioned time limits were confirmed as long enough for these groups in a pilot study. The paradigm was presented on a desktop computer using Presentation^®^ (Neurobehavioral Systems Inc., San Francisco, CA, USA).

#### Interference Task

In the EI and the LI sessions, pictures of natural and artificial objects from a different set than in the encoding session were shown in the middle of the screen consecutively with an interval of 1.3 s. Participants were asked to press one assigned button, whenever three natural or three artificial objects occurred in a row. The task contained five blocks of 30 pictures with three target sequences in each round. At the end of each block, a fixation cross was shown for 30.9 s. The performance was measured as hits in relation to the number of targets. In the NI session, participants rested in the test room without any distracting influences. They were asked to remain silent and seated, which was controlled by observation.

### Statistical Analyses

Analysis was conducted using SPSS 21 (IBM Corp. Released 2012. IBM SPSS Statistics for Windows, Version 21.0. Armonk, NY, USA: IBM Corp.). We calculated the percentage of correctly recognized (object recognition) and positioned objects (fine- and coarse-grained spatial memory), separately. To assess the main effect of interference irrespective of timing, we averaged the results of both interference sessions. In order to validate the different effects of interferences between all groups and to test for any interaction, we used a mixed factor analysis of variance (ANCOVA, Factor 1: Group, Factor 2: Interference, covariate of no interest: years of education). We further processed ANCOVAs on the different interferences to check for differences between EI and LI. For each group of participants (young, old, aMCI), between-session differences were computed using paired *t*-tests. Between-group comparisons were performed using unpaired *t*-tests. For the interference task, we applied a signal detection analysis to investigate group differences (Green and Swets, 1996). The sensitivity index *d*′ computes the distance between the signal (hit) and noise (false alarm) distribution means in standard deviation units. The parameter was calculated as described in Stanislaw and Todorov (1999) and Kukolja et al. (2009): *d*′ = Φ − 1(*H*′) − Φ − 1(*F*′) where *H*′ is the corrected hit rate, *F*′ the corrected false alarm rate, and Φ − 1 is the function converting probabilities into *z* scores. To protect against ceiling effects with *H* of 1 and *F* of 0 (corresponding *z*-values would be +∞ or −∞, respectively), we used corrected values of *H* and *F*: *H*′ = (*h* + 0.5)/(*h* + *m* + 1) and *F*′ = (*f* + 0.5)/(*f* + *cr* + 1), where *h* is the number of hits, *m* the number of misses, *f* the number of false alarms, and *cr* is the number of correct rejections. Group comparisons were performed by an ANCOVA. The alpha level was set to *p* < 0.05 for all analyses.

## Results

### Demographic and Neuropsychological Measures

All subjects with aMCI fulfilled the criteria according to Petersen (Petersen, 2004). The average number of formal years of education was 13.78 years for the young controls, 13.00 years for the older controls, and 11.92 years for the aMCI-subjects. The difference between young controls and aMCI-subjects was significant (unpaired *t*-test, *p* = 0.016), whereas the differences between young and older controls and between older controls and aMCI-subjects were not significant. Accordingly, this variable was used as a covariate of no interest in the respective tests (as described in the “Statistical Analyses” section). As presented in Table 1, the aMCI-group showed deficits of verbal memory, depicted by a poorer performance more than 1.5 standard deviations below the norm on the delayed recall in the VLMT, but were within the lower normal range in tests of short-term memory and other cognitive functions, and did not complain of constraints in activities of daily living (measured by Bayer-ADL). All controls showed normal IQ-rates and sufficient performance of short- and long-term memory tasks.

**Table 1:** Neuropsychological measures for the three groups.

	Young controls (20 m/19 f)		Old controls (20 m/19 f)		Patients (7 m/5 f)		
	Mean	SD	Mean	SD	Mean	SD	*p*
Rey-Osterrieth CFT (T)	52.87	6.25	55.46	4.27	49.08	13.70	n.s.
VLMT (A7—late recall)	13.21	2.11	11.49	2.14	3.58	2.07	***
Edinburgh decile	R61.26	47.75	R72.50	41.61	R79.25	31.00	n.s.
BDI V	19.28	10.73	20.03	9.88	23.42	15.66	n.s.
BTA	17.59	2.41	17.82	2.05	15.58	4.10	n.s.
Mac- Q	19.74	5.21	22.29	5.35	25.67	7.06	*
Bayer-ADL	1.38	0.36	1.40	0.41	2.46	1.84	n.s.
TMT (A)	25.72	7.24	44.26	12.17	42.08	13.47	n.s.
TMT (B)	51.10	15.60	90.26	28.83	143.75	82.37	*
LPS-4 (C)	7.90	1.48	6.41	1.48	4.92	2.07	**
DemTect	17.31	1.28	15.95	2.04	12.17	3.24	***

The Complex-Figure-Test (CFT) by Rey-Osterrieth is from Pena-Casanova et al. (2009); The Verbal Learning and Memory Test (VLMT) is from Lux et al. (1999); the Edinburgh handedness inventory is from McMeekan and Lishman (1975); the Becks depression inventory (BDI V) is from Beck and Steer (1984); the Brief test of attention (BTA) is from Schretlen et al. (1996); the Memory Assessment Clinics Questionnaire (the MAC-Q) is from Crook et al. (1992); the Bayer Activities of daily Living Scale (Bayer-ADL) is from Hindmarch et al. (1998); the Trail-making Test (TMT A and B) is from Reitan (1955); the Leistungsprüfsystem, test 4 (LPS-4) is from Horn and Cattell (1966); and the Demenz-Detektion (DemTect) is from Kalbe et al. (2004). *p < 0.05, **p < 0.01, ***p < 0.001, n.s., not significant.

### Level of Task Difficulty

Based upon the baseline session performance, young participants were assigned to task difficulty levels 1 to 4 (mean 2.00, median 2), older participants were assigned to levels 2 to 5 (mean 3.26, median 3), and aMCI-subjects were assigned to levels 3 to 6 (mean 4.33, median 4). As to be expected, the assigned level of task difficulty differed significantly between groups (paired *t*-test, *p* < 0.001 for young vs. older adults and young vs. aMCI-subjects, respectively, and *p* < 0.01 for older adults vs. aMCI-subjects).

### Interference Task Performance

The interference task was designed to interfere with consolidation processes. The sensitivity index *d*′ was computed to analyze the distance between the signal and noise distribution means in standard deviation units. A significant difference between the groups could not be detected (*d*′ for the young group: 3.29 ± 0.53; *d*′ for the older group: 3.12 ± 0.94; *d*′ for the MCI-group: 3.34 ± 0.90).

### Effect of Interference

#### Effect of Timing of Interference

**Figure 3:**
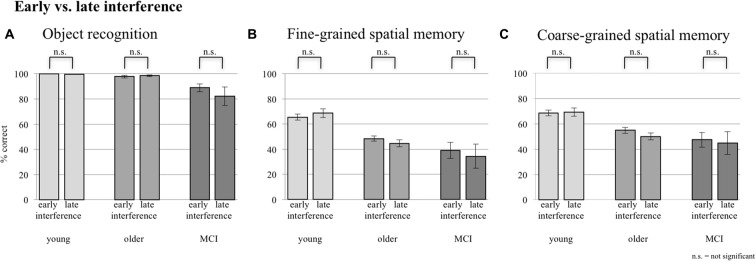
Comparison between early and late interference (EI and LI) for all three groups concerning **(A)** object recognition, **(B)** fine-grained spatial memory and **(C)** fine- + coarse-grained spatial memory. Error bars represent the standard error.

A comparison of performance levels of the sessions with EI and the ones with LI did not reveal any significant difference in any group for neither object memory, fine-grained spatial memory, nor coarse-grained spatial memory (see Figure 3).

As EI and LI did not reveal any significant differences, data of both interference sessions were pooled to assess the main effect of interference for the following analyses.

#### Object Memory

Young subjects achieved 99.82% ± 0.79 correct answers for object memory in the sessions with and 99.82% ± 1.12 without interference (paired *t*-test, *p* = 1.0). In comparison, older controls achieved 98.41% ± 4.73 correct answers without interference and 98.09% ± 4.91 with interference (paired *t*-test, *p* = 0.639). Thus, in both groups no effect of interference was observed. In contrast, the aMCI-group showed a decline of recognition of previously seen objects due to interference. aMCI-subjects achieved 95.58% ± 6.93 correct answers without interference and 85.53% ± 19.08 with interference (paired *t*-test, *p* = 0.030). An ANCOVA (factor group and factor interference; covariate years of education) revealed that this performance loss was significant at the group level (*F*_(2,86)_ = 18.294, *p* < 0.001). This effect was primarily driven by the difference between the aMCI-group and the control groups, as the difference between the two control groups was not significant (*F*_(1, 75)_ = 0.398, *p* = 0.530).

#### Fine-Grained Spatial Memory

**Figure 4:**
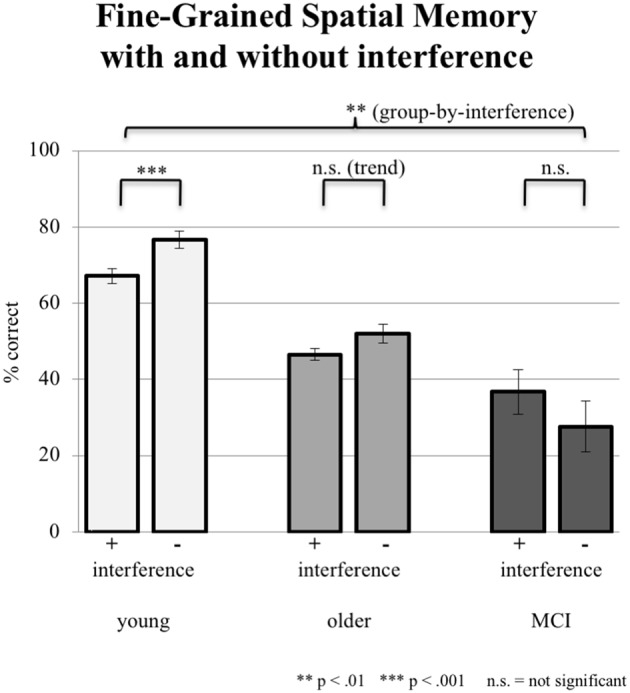
Correct responses in % depicted for all three groups under the condition of interference (pooled data from EI and LI) during consolidation vs. no interference (NI). Error bars represent the standard error.

In fine-grained spatial contextual memory, young participants performed better than elderly participants, yet the latter performed better than subjects with aMCI in allocating recognized objects in the grid. The ANCOVA (factor group, factor interference mode; covariate years of education) revealed a significant group-by-interference effect (*F*_(2,86)_ = 4.926, *p* = 0.009; see Figure 4). Young subjects were significantly disturbed by interference (76.67% ± 13.62 correct answers during sessions without interference and 67.13% ± 17.71 during sessions with interference; paired *t*-test, *p* = 0.001). Older controls also showed deterioration in performance (from 52.08% 15.50 without to 46.53% ± 13.76 with interference). However, this difference revealed only a trend toward significance (paired *t*-test, *p* = 0.087). Finally, aMCI-subjects did not show an effect of interference (27.58% ± 23.24 without and 36.75% ± 28.61 with interference, paired *t*-test, *p* = 0.218). Mean performance levels were above chance level as tested by a binominal test.

#### Coarse-Grained Spatial Memory

**Figure 5:**
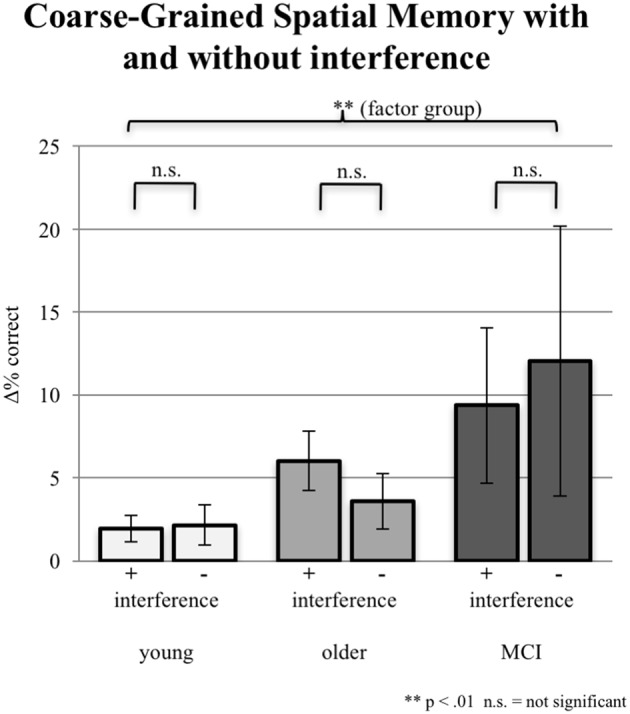
Imprecise correct responses (one field next to the original position either horizontally or vertically) in % for all three groups under the condition of interference (pooled data from EI and LI) during consolidation vs. the condition of NI. Note that precise correct responses are not included. Error bars represent the standard error.

In addition to the exact correct positioning presented in the section “Fine-Grained Spatial Memory,” young participants accomplished additional 2.14% coarse-grained positioning of the objects in the no-interference session and 1.93% in the interference sessions. Older participants reached additionally 3.56% coarse-grained positioning without interference and 6.01% with interference. aMCI-subjects achieved the highest proportion of coarse-grained positioning: 9.36% with interference; 12.04% without interference. Differences between interference and no-interference sessions were not significant within groups. The ANCOVA (factor 1 group, factor 2 interference; covariate years of education) testing for an interaction effect was not significant (*F*_(2,86)_ = 0.513, *p* = 0.600). However, the factor group (irrespective of interference) revealed a significant group effect (*F*_(2,86)_ = 5.636, *p* = 0.005; see Figure 5).

**Figure 6:**
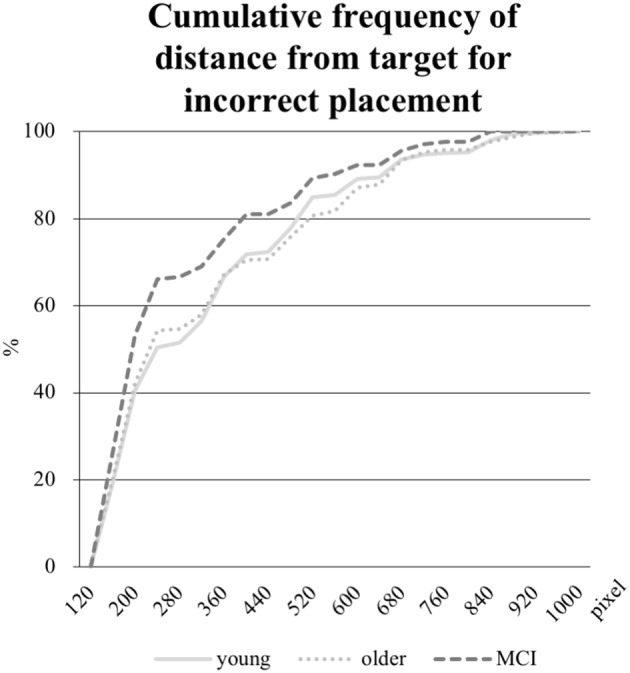
Cumulative frequency in percent of distances in pixel from the object placement within the grid to the center of the target field (edge length for every field is 160 pixel). Only the distances from incorrectly placed objects are analyzed.

In addition, the mean distance in pixel of the object placement within the grid by the participant from the center of the target field was calculated for the objects incorrectly positioned. Whereas the mean distance was 326 pixels in the young group and 321 pixels in the older group, the aMCI-group placed the objects 274 pixels away from the target. As the Kruskal-Wallis test revealed an unequal distribution, a Mann-Whitney-U-Test was performed to test for significance. The difference between the young and the older group was not significant, however, both the comparisons between the young and the aMCI-group (*p* = 0.017) and between the older and the aMCI-group (*p* = 0.018) were significant. Further, we computed the distribution of the distances (see Figure 6), which reveals a shift towards shorter distances in the aMCI-group in contrast to the young and older group.

#### Relationship Between Task Performance and Neuropsychological Testing

In order to test whether the task performance and the memory based neuropsychological tests VLMT and CFT are correlated, we performed Pearson’s correlation between these measures. Significant associations could be detected for all combinations between the object recognition/spatial context and all sub-scores of the VLMT and the CFT (r-values differ from 0.303 to 0.672, *p* < 0.01), the only combination not showing a significant result was between the spatial context and CFT (*r* = 0.167, *p* = 0.112).

## Discussion

In this study, we investigated the effect of interference during consolidation of objects and their associated spatial position in healthy young and older subjects as well as subjects suffering from aMCI. The main results of this study are: (i) a negative effect of interference on consolidation of spatial memory in healthy young subjects and to a lesser degree in older healthy subjects, but not in aMCI-patients; (ii) a shift from fine- to coarse-grained spatial representation in the aMCI-group; and (iii) no differential effect of EI in comparison to LI in either of the groups.

### Effect of Interference

Interference during consolidation evoked differential group-specific effects on spatial memory. A significant negative interference effect could be detected in the young group. Older subjects also showed a negative effect, albeit to a lesser degree with only a trend toward significance. Following a continuum from a strong negative over a weak negative to a missing effect, aMCI-subjects showed no effect of interference at all despite the fact that the above chance performance demonstrated encoding. A different pattern emerged when analyzing object memory irrespective of spatial context. An impaired object memory was observed for the aMCI-group in the interference sessions in relation to the NI session, in which the young and the older group showed a ceiling effect without and with interference. As aMCI-subjects showed an adequate performance in the interference task, this finding demonstrates that the interference task engaged the aMCI-group on a cognitive level and disturbed consolidation of object memory but not spatial memory. The fact that one memory category was disrupted whereas the other was unaffected suggests that this effect is not an unspecific effect of working memory in terms of, e.g., a simple overload.

One possible explanation for the differential effect might be that consolidation processes for object vs. spatial memory differ from each other in the aMCI-group and that distinct networks are engaged (Moscovitch et al., 1995). Studies with healthy subjects have shown that object and spatial memory differentially activate the fusiform gyrus (Kukolja et al., 2009) and the supramarginal gyrus (Moscovitch et al., 1995), indicating a differential involvement of ventral and dorsal pathways. Khan et al. (2013) presumed that especially the anterior pathway is affected in early stages of disease, as neuroimaging shows a decreased cerebral blood volume in the parahippocampal gyrus and the entorhinal cortex already in patients with preclinical Alzheimer’s disease. In addition, this area has shown to be involved in spatial memory (Burgess et al., 2001).

Taking this into account, one could argue that spatial memory consolidation is mediated by hippocampus-dependent processes in the healthy condition and that in the course of neurodegeneration and hippocampal dysfunction a compensatory shift to less hippocampus-dependent processes takes place. Another explanation might be, that in functionally intact conditions (i.e., in healthy subjects), spatial memory consolidation is mediated by distinct functions of hippocampus-subunits (e.g., the differentiation of anterior and posterior hippocampus functionality) and that in the course of aging, neurodegeneration, and hippocampal dysfunction a de-differentiation takes place and, hence, an interference causes differential effects whether the subunits function or not. Recent research indicates different pathways in the hippocampal region for different types of memories. In a study using neuroimaging techniques, Reagh and Yassa (2014) were able to show, that a combined object and spatial memory task activates two parallel but interacting networks encompassing the hippocampus. Furthermore, Fidalgo et al. (2016) have pointed out a stronger impairment in object than scene memory in aMCI-patients, and a higher predictive value of impaired object memory, whilst perirhinal cortex and lateral entorhinal cortex are supposed to process object information. According to this, neuroimaging shows a decreasing domain-specific activity in the perirhinal cortex (Berron et al., 2018).

Besides the fact that object memory and spatial memory draw upon different types of information, another difference may underlie the observed differential interference effect. In the applied paradigm, object memory was purely based upon recognition (Squire et al., 2004; Hanseeuw et al., 2012) whereas spatial allocation of objects needed active recall of spatial contextual information (Squire et al., 2007). Notoriously, recall demands different processing resources than recognition (Craik and Mcdowd, 1987). Accordingly, studies often show preserved recognition, whilst recall is more often impaired in amnesic patients (Reed et al., 1997), moreover, in patients even decades before the clinical onset of dementia (Elias et al., 2000). A comparison of impaired and unimpaired elderly subjects showed an overall decline of object memory with age, whereas spatial context only declined in the already impaired group (Reagh et al., 2016). Wang et al. (2013) were able to demonstrate that a decline of context spatial memory is a valid indicator for ongoing aMCI. Our findings for a relatively preserved object memory in aMCI-patients are consistent with this.

### Fine- vs. Coarse-Grained Spatial Representation

Spatial memory can be divided into fine-grained representations, which store spatial coordinates, and coarser representations, which store spatial information categorically (Moscovitch et al., 2006). The latter reflects a natural partitioning of the environment, referring the to-be-retained material to, e.g., a quadrant in the field of view (Fitting et al., 2009). Moreover, studies showed that the accuracy of spatial information fades with time (Badcock et al., 2008). In our study, young subjects remembered the position of objects either exactly or not at all. In contrast, aMCI-subjects retrieved the position more vaguely in a considerable number of trials, as hypothesized. Healthy older subjects showed again an intermediate performance between young subjects and aMCI-subjects. Thus, data suggest that in the course of aging and neurodegeneration, a shift from fine- to coarse-grained spatial representation is observable. Thus, coarse-grained representations may be less dependent upon the hippocampus since they are processed in the parahippocampal gyrus, lingual gyrus, posterior cingulate cortex, retrosplenial and lateral temporal cortex (Rosenbaum et al., 2004, 2007; Hirshhorn et al., 2012), whereas storing exact spatial information is associated with increased neural activity in the posterior hippocampus (Nadel et al., 2013). Further evidence for this notion is provided by studies investigating patients with hippocampal lesions: it has been shown that fine-grained spatial details get lost while coarse information can still be retrieved (Rosenbaum et al., 2000).

### Effects of the Timing of Interference

As a final aspect of the analysis, we were interested whether timing of interference had an impact on the investigated memory processes, as has been suggested (Skaggs, 1925; Newton and Wickens, 1956). In a study by Dewar et al. (2009) it was shown that episodic memory consolidation is most vulnerable to interference in aMCI-subjects when compared to controls, and that this effect was present during the initial phases of consolidation, but not at later stages. This time dependency was not observed in healthy controls (Dewar et al., 2009). Similar effects were not observed in our study. However, in comparison to the above-mentioned study, our study differs in several relevant aspects. The main difference is that we tested spatial memory and object memory whereas Dewar et al. (2009) performed a word-list task. In addition, the here applied interference task drew upon working memory whereas Dewar et al. (2009) used a picture-naming-task with superimposed words, which were partly incongruent to the pictures; and finally, albeit probably less relevant, we used two blocks of 6 min instead of three blocks of 3 min for the interval between encoding and retrieval.

### Limitations

The small number of subjects in the aMCI-group and the high variance in relation to the two other groups is one of the limitations of the study; a replication with a larger patient-group is needed. Further, we aimed at similar performance levels across the three groups (±25% between groups) and the absence of ceiling and floor effects (25%–75% correct response). With administration of different individual difficulty levels, we accepted differences with regard to the number of objects to-be-learned and repetitions of the same object shown. Although, we have taken great effort in piloting the paradigm and adjusting individual difficulty levels, it was not possible to achieve similar performance levels for object and spatial memory likewise. As our hypotheses were based on spatial memory processes, we adjusted the paradigm according to the latter. Our main aim to prevent ceiling and floor effects in spatial memory was achieved. All the performance levels acquired were in a valid range and far beyond the chance level of 5% concerning spatial memory. The ceiling effect concerning object memory for the young and older group was not preventable without jeopardizing performance levels in spatial memory as determined during pilot testing. Another limitation might be an unspecific effect of the interference task. Although the interference task itself differed from the encoding and retrieval tasks, the stimuli were very similar in nature to those presented during encoding and retrieval. This might have caused retrieval interference in addition to consolidation interference (Wixted, 2004). Since retrieval interference is strongest when similar items are placed immediately after encoding or immediately prior to retrieval, this might explain why an effect of timing of interference was washed out. Finally, in this study only the very early period of consolidation could be investigated. The classic view of consolidation is that the hippocampal system and the relevant neocortical areas are responsible for initial processing; with increasing time, the memory traces shift to neocortical networks, thereby becoming increasingly independent from the hippocampal formation (Piefke et al., 2003; Takashima et al., 2009; Smith et al., 2010). According to an alternative model, the hippocampus-neocortical system encodes distributed traces. With recurrent reactivations, multiple hippocampal-dependent traces exist. Therefore, this multiple trace theory suggests that the hippocampus remains continuously involved in the storage and retrieval of episodic memories (Nadel et al., 2000). Both models assume that memory organization happens in an interplay between the hippocampus and distinct neocortical areas, however, whether or not the links with the hippocampus fade away or are continuously involved differs (Nadel and Bohbot, 2001; Dudai, 2004; Nadel et al., 2012). As the focus of this study was the (very) early period of consolidation and as it is assumed that the difference between both models becomes relevant after a certain period of time, inferences on how interference affects consolidation after a longer period of consolidation cannot be made.

## Conclusion

Altogether, our findings imply that the consolidation process for spatial information in subjects with aMCI differs from the one in healthy individuals. Under the condition of impaired memory (and hippocampal dysfunctioning), object memory is vulnerable to interference, whilst spatial memory does not seem to be disturbed further. This may be due to a de-differentiation of hippocampus-subunit-connectivity in aging and neurodegeneration. Studies using functional imaging may elucidate the neural basis for this observation and may find explanations for the shift from fine-grained to coarse-grained spatial representation. This preserved function of coarse-grained spatial representation may be a basis for compensatory mechanisms and a target to train individuals suffering from aMCI to preserve independency in daily living.

## Data Availability

Datasets are available on request: the raw data supporting the conclusions of this manuscript will be made available by the authors, without undue reservation, to any qualified researcher.

## Author Contributions

All authors listed in the author list fully qualify for authorship, all authors contributed significantly to the work. OO, HM, GF and JK designed the experiment. HM collected the data. HM, NR, BR, JK and OO analyzed the data. HM, NR, BR, JK, GF and OO wrote, revised and approved the manuscript.

## Conflict of Interest Statement

The authors declare that the research was conducted in the absence of any commercial or financial relationships that could be construed as a potential conflict of interest.
